# P-509. Gestational Lyme disease incidence and pregnancy-related outcomes among women in the United States, 2015-2024

**DOI:** 10.1093/ofid/ofaf695.724

**Published:** 2026-01-11

**Authors:** Sarah J Willis, Julie Davis, Tiange Yu, Pulkit Sehgal, L Hannah Gould, Stephanie Duench, Ye Tan, James H Stark, Sarah B Mulkey, Roberta L DeBiasi

**Affiliations:** Pfizer, Wakefield, Massachusetts; Pfizer, Wakefield, Massachusetts; Genesis Research Group, Hoboken, NewJersey; Genesis Research Group, Hoboken, NewJersey; Pfizer Vaccines, New York, New York; Pfizer, Inc., Collegeville, Pennsylvania; Pfizer Inc., Cambridge, Massachusetts; Pfizer Biopharma Group, Collegeville, Pennsylvania; Children's National Hospital/ George Washington University School of Medicine and Health Sciences, Washington, DC; Children's National Hospital/The George Washington University School of Medicine and Health Sciences, Washington, District of Columbia

## Abstract

**Background:**

Gestational Lyme disease (LD) case reports describe adverse outcomes ranging in severity from a mild rash to intrauterine fetal death, cortical blindness, prematurity and syndactyly. We estimated gestational LD incidence and frequency of adverse outcomes among pregnancies with and without LD using a large administrative claims database.

**Table.**
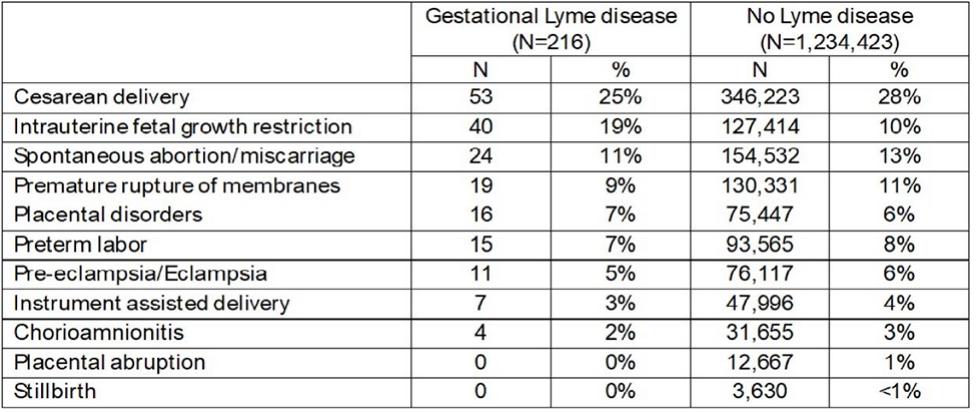
Pregnancy related outcomes among pregnancies with and without gestational Lyme disease, 2015 - 2024

**Methods:**

We identified pregnant women 12–55 years between October 2015-December 2024 in Optum® Clinformatics® Data Mart. A validated claims-based gestational age algorithm estimated pregnancy start and end dates. Gestational LD was defined as ≥1 LD diagnosis code and, for outpatient diagnoses, ≥7 days oral or ≥1 intravenous LD antibiotic ±30 days. We captured gestational LD < 30 days from pregnancy start date through pregnancy end date. We estimated the incidence of gestational LD per 100,000 pregnancies between 2016-2023 [calendar years with complete data] and measured adverse pregnancy outcomes using diagnosis/procedure codes among pregnancies with and without gestational LD.

**Results:**

We identified 1,234,639 pregnancies between October 2015 and December 2024 and 216 (0.02%) with gestational LD. Age, race, and ethnicity were similar across all pregnancies. Pregnant women with LD were more likely to reside in a high-incidence LD state (63% vs. 23% without LD).

Average annual incidence of gestational LD was 18.1 cases per 100,000 pregnancies (range: 13.3 to 24.1 cases per 100,000 pregnancies). Average annual incidence was higher among pregnant women residing in high-incidence LD states than low-incidence LD states (50.8 vs. 7.6 cases per 100,000 pregnancies).

Similar proportions of pregnancies with and without LD resulted in spontaneous abortion, PROM, pre-eclampsia, chorioamnionitis, placental abruption, preterm labor, instrument assisted delivery, stillbirth, and placental disorders (Table). However, a greater proportion of pregnancies with LD experienced intrauterine fetal growth restriction (19% vs 10%) and fewer had cesarean deliveries (25% vs. 28%).

**Conclusion:**

The frequencies of most adverse pregnancy outcomes were similar among pregnancies with and without LD. However, pregnant women who reside in or travel to endemic regions of the US should prioritize preventive LD measures.

**Disclosures:**

Sarah J. Willis, PhD, MPH, Pfizer, Inc.: Stocks/Bonds (Public Company) Tiange Yu, MS, Pfizer Inc.: Employee of Genesis Research Group, which has received consulting fees from Pfizer Inc. Pulkit Sehgal, n/a, Pfizer Inc.: Pfizer Inc. (Employee of Genesis Research Group, which has received consulting fees from Pfizer Inc.) L. Hannah Gould, PhD, MS, MBA, Pfizer Inc.: Employee; may hold company shares and/or stocks. Stephanie Duench, PhD, Pfizer, Inc.: Employment|Pfizer, Inc.: Stocks/Bonds (Public Company) James H. Stark, PhD, Pfizer Vaccines: Employer|Pfizer Vaccines: Stocks/Bonds (Public Company) Sarah B. Mulkey, MD, PhD, Pfizer: Advisor/Consultant Roberta L. DeBiasi, MD, MS, Pfizer: Grant/Research Support

